# Cohort profile: the China surgery and anesthesia cohort (CSAC)

**DOI:** 10.1007/s10654-023-01083-4

**Published:** 2024-01-10

**Authors:** Lei Yang, Wenwen Chen, Dongxu Chen, Junhui He, Junren Wang, Yuanyuan Qu, Yao Yang, Yuling Tang, Huolin Zeng, Wanxin Deng, Hongxin Liu, Lining Huang, Xuze Li, Lei Du, Jin Liu, Qian Li, Huan Song

**Affiliations:** 1 Department of Anesthesiology and West China Biomedical Big Data Center, West China Hospital, Sichuan University, Guo Xue Lane 37, Chengdu, China; 2 West China Biomedical Big Data Center, West China Hospital, Sichuan University, Guo Xue Lane 37, Chengdu, China; 3 Med-X Center for Informatics, Sichuan University, Chengdu, China; 4 Department of Anesthesiology, West China Hospital, Sichuan University, Chengdu, China; 5 Surgical Anesthesia Center, The First People’s Hospital of Longquanyi District, Chengdu, China; 6 Department of Anesthesiology, The Second Hospital of Hebei Medical University, Shijiazhuang, China; 7 Center of Public Health Sciences, Faculty of Medicine, University of Iceland, Reykjavík, Iceland; 8 Institute of Environmental Medicine, Karolinska Institute, Stockholm, Sweden

**Keywords:** Cohort study, Surgery, Anaesthesia, Middle-aged

## Abstract

The China Surgery and Anaesthesia Cohort (CSAC) study was launched in July 2020 and is an ongoing prospective cohort study recruiting patients aged 40–65 years who underwent elective surgeries with general anaesthesia across four medical centres in China. The general objective of the CSAC study is to improve our understanding of the complex interaction between environmental and genetic components as well as to determine their effects on a wide range of interested surgery/anaesthesia-related outcomes. To achieve this goal, we collected enriched phenotypic data, e.g., sociodemographic characteristics, lifestyle factors, perioperative neuropsychological changes, anaesthesia- and surgery-related complications, and medical conditions, at recruitment, as well as through both active (at 1, 3, 7 days and 1, 3, 6, 12 months after surgery) and passive (for more than 1 year after surgery) follow-up assessments. We also obtained omics data from blood samples. In addition, COVID-19-related information was collected from all participants since January 2023, immediately after COVID-19 restrictions were eased in China. As of July 18, 2023, 12,766 participants (mean age = 52.40 years, 57.93% were female) completed baseline data collection (response rate = 94.68%), among which approximately 70% donated blood and hair samples. The follow-up rates within 12 months after surgery were > 92%. Our initial analyses have demonstrated the incidence of and risk factors for chronic postsurgical pain (CPSP) and postoperative cognitive dysfunction (POCD) among middle-aged Chinese individuals, which may prompt further mechanistic exploration and facilitate the development of effective interventions for preventing those conditions. Additional studies, such as genome-wide association analyses for identifying the genetic determinants of CPSP and POCD, are ongoing, and their findings will be released in the future.

**Supplementary Information:**

The online version contains supplementary material available at 10.1007/s10654-023-01083-4.

## Introduction

Approximately 321.5 million surgeries were performed worldwide in 2010, and this incidence continues to grow due to the increase in affordable and accessible medical care services, especially in developing countries [1]. A European cohort study reported an in-hospital postoperative mortality rate of up to 4.0% [2]. Furthermore, postoperative mortality within 30 days accounts for 7.7% of all deaths globally and is ranked as the third leading cause of death worldwide [3]. Notably, in addition to the deterioration of the index disease per se (e.g., cancer, pancreatitis, valvulopathy), adversities or complications that are related to surgery and anaesthesia have also contributed to the high morbidity and mortality rate during the time period directly after such an experience, which results in a decreased quality of life after surgery and an increased disease burden among this vulnerable population [4, 5]. Therefore, it is necessary to improve postoperative safety by optimizing perioperative interventions, which is a key and important task for global surgeons and anaesthesiologists.

Nevertheless, only a handful of studies have examined surgery- or anaesthesia-related consequences, and most of them have focused on selected severe postoperative complications, within-hospital mortality, health resource use, and postoperative recovery. Other relevant outcomes with potential clinical importance (e.g., chronic postoperative pain, cognitive decline, psychological conditions) remain less explored [6]. Moreover, previous investigations usually have methodological shortcomings, such as limited sample size [7], retrospectively collected data [8], insufficient controls for somatic or psychological conditions [9], and inaccurate measurement of exposures of interest [10].

### General and specific objectives

The China Surgery and Anaesthesia Cohort (CSAC) was launched in 2020, and its general objective is to provide a reliable source of data and detailed perioperative multidimensional information (e.g., lifestyle factors, somatic conditions, neuropsychological conditions, genetic information, and anaesthesia-related information, including intraoperative vital signs and events). This information can enhance knowledge regarding the complex interplay of environmental and genetic components as well as their effects on a wide range of interested surgery/anaesthesia-related outcomes, with a primary focus on modifiable factors (e.g., perioperative neuropsychological conditions). Particularly, in December 2022, the Chinese government announced that COVID-19 restrictions would be eased, which led to a tremendous increase in COVID-19 cases in the cities where our included medical centres were located. We therefore additionally collected relevant data about COVID-19 infection from all recruited participants since January 2023, thus providing a unique opportunity for studying phenotypic and genotypic risk factors associated with COVID-19 symptoms and severity among the Chinese surgical population [11]. Our specific objectives include but are not limited to:

- Describe the incidence of surgery/anaesthesia-related adversities or complications that occurred in the hospital (1, 3, and 7 days after the surgery) or during follow-up (1, 3, 6, and 12 months after the surgery) and provide data supporting the development of health-related policy.
- Disentangle the impacts of perioperative neuropsychological conditions and COVID-19 infection on multiple surgery/anaesthesia-related outcomes and elucidate the potential mechanisms using omics data extracted from biological samples.
- Clarify the influence of surgery/anaesthesia-related conditions on both short- and long-term health consequences with a primary focus on chronic postsurgical pain (CPSP) and postoperative cognitive dysfunction (POCD), and determine whether such a risk can be modified by the individuals’ genetic susceptibility.
- Explore the genetic basis of multiple surgery/anaesthesia-related adversities or complications, including malignant hyperthermia, postoperative neurocognitive disorder, and postoperative pain (acute and chronic).
- Investigate phenotypic and genotypic risk factors that are associated with susceptibility to and the severity of COVID-19 among middle-aged Chinese individuals.

In the present article, we described the objectives, methodology, key findings, main strengths and limitations, and perspectives of the CSAC.

## Methods

### Study design and population

The CSAC is an ongoing multicentre prospective cohort study in China that was first established in West China Hospital in July 2020 and then gradually expanded to three additional medical centres (The First People’s Hospital of Longquanyi District since August 2021, West China Tianfu Hospital since May 2022, and The Second Hospital of Hebei Medical University since September 2022). The initial focus of the study was noncardiac surgeries, such as abdominal, thoracic or otorhinolaryngologic surgery; cardiac surgeries were included starting in July 2021.

Based on the age distribution of the population at risk of surgery with anaesthesia, as well as the reported incidence of surgical/anaesthesia complications [2, 12–14] and the major disease outcomes of interest (e.g., psychiatric disorders and neurodegenerative diseases) among the Chinese population [15], we aimed to recruit all patients aged 40–65 years who underwent elective surgeries with general anaesthesia in the included medical centres and who provided blood and hair samples. Therefore, the inclusion criteria were as follows: (1) patients aged between 40 and 65 years; (2) patients who planned to receive elective surgery; and (3) patients who agreed to receive general anaesthesia during the operation. The exclusion criteria included patients who were not residents of the city where the recruitment medical centre was located, patients who received day surgeries (the length of hospital admission < 2 days) or craniotomy, patients who had an education level less than primary school, and patients who were unable to understand the interview scales. The pilot study was conducted between January 1st and July 14th, 2020 in West China Hospital, during which we developed a customized pipeline for optimizing the standardized procedure of data and biosample collection and quality control.

### Data collection

#### Baseline information

**Fig. 1:**
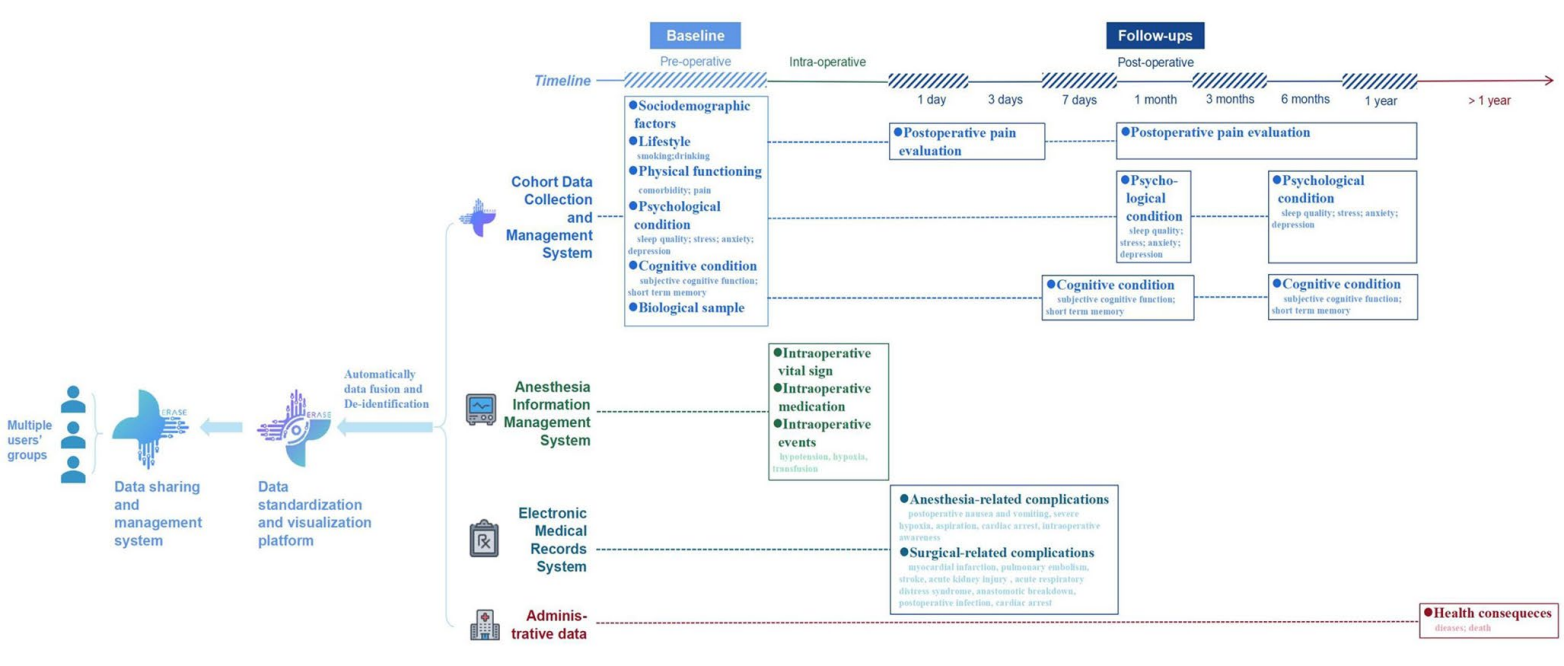
The data framework of the China Surgery and Anesthesia Cohort (CSAC)

As shown in Fig. 1, data about sociodemographic factors, lifestyle factors, physical functioning, and preoperative psychological and cognitive conditions were collected through face-to-face interviews conducted by well-trained data collectors (training course checklist is shown in supplementary materials 1) at baseline (i.e., one day before surgery date) using touchscreen questionnaires implemented in a newly developed Cohort Data Collection and Management System (CD-CMS) (Version 1.0, Build 2021SR0484324. ©West China Hospital, Sichuan, China). Biological samples were collected on the day of surgery prior to anaesthesia.

#### Follow-ups

At least two phone numbers of the patient and/or their relatives were saved at initial recruitment for further contacts. Well-trained data collectors scheduled active follow-ups for each participant within 1 year (Fig. 1) using the abovementioned data collection system. Specifically, the face-to-face follow-ups on days 1, 3 and 7 after the operation mainly focused on assessing in-hospital anaesthesia and surgical-related complications, postoperative pain, and immediate changes in cognitive condition. The follow-ups after hospital discharge focused on measuring the trajectories of postoperative pain and cognitive and psychological conditions; follow-up assessments were conducted through telephone interviews or online questionnaires at 1 month, 3 months, 6 months and 1 year after the surgery. In practice, we could allow ± 1 week for these scheduled follow-ups, and unless the participants withdrew their informed consent or refused further contacts, we continued subsequent follow-ups even if the participants have missed the previous one(s). Additionally, to ensure high follow-up rates, data collectors reviewed the participants’ baseline records before calling them, in order to customize some specific questions and provide disease-related advice. We also offered to help participants with reaching their doctors/other specialists if needed.

Health consequences after 1 year are planned to be obtained by periodically linking the dataset to established administrative registers. Specifically, the linked data for diseases diagnosed 1 year after surgery will be obtained from the National Health Insurance claim databases where the medical diagnoses were documented according to the 10th version of the International Statistical Classification of Diseases (ICD-10). In addition, the underlying and contributing causes of death will be derived based on the death certificates reported to the regional mortality registries.

#### Other data resources

The CSAC study also included enriched clinical data through linkage with several electronic medical data resources (Fig. 1). For instance, details related to the surgery and general anaesthesia procedure were documented in the anaesthesia information management system (AIMS), and thus, our database has enriched variables on intraoperative events (e.g., hypotension, hypoxia, transfusion), dynamically measured vital signs, and medication and anaesthesia drug use. Information about disease diagnoses, the results of medical examination (laboratory tests and imaging examination), and medical care received in the hospital was obtained from the electronic medical record (EMR) system of each recruitment centre.

We summarized the variables available at each time point, as well as their measurement approaches and resources, in Supplementary Table 1.

#### Measurement of primary outcomes and conditions

Neuropsychological symptoms at each time point were assessed by several well-validated scales. Sleep quality was measured by the Pittsburgh Sleep Quality Index [PSQI] [16]. Stress was measured by the Impact of Event Scale-Revised version [IES-R] [17] and Posttraumatic Stress Disorder Checklist for DSM-5 [PCL-5] [18]. Anxiety was measured by the Generalized Anxiety Disorder 7-Item Scale [GAD-7] [19]. Depression was measured by the Patient Health Questionnaire [PHQ-9] [20]. Subjective cognitive function was measured by the Eight-item Informant Interview to Differentiate Aging and Dementia [AD-8] [21]. Short-term memory was measured with the three-word recall test [22]. Self-evaluated pain level was measured by the Brief Pain Inventory [BPI] [23]. We then determined the presence of those conditions using the cut-off points that have been validated in the Chinese population (Supplementary Table 2).

The medical records were manually reviewed by the trained data collector to determine the presence of postoperative complications of interest, including anaesthesia-related complications (i.e., postoperative nausea and vomiting, severe hypoxia, aspiration, cardiac arrest and intraoperative awareness) and surgery-related complications (i.e., myocardial infarction, pulmonary embolism [PE], stroke, acute kidney injury [AKI], acute respiratory distress syndrome [ARDS], anastomotic breakdown, postoperative infection, and cardiac arrest), in accordance with European Perioperative Clinical Outcome (EPCO) definitions [24].

On December 7, 2022, the Chinese government announced rollback of strict anti-COVID-19 measures [25]. We recontacted all participants to obtain data related to COVID-19 between January and April 2023, including vaccination history, infection status, diagnosis methods, symptoms (type, severity, and duration) and treatment (Supplementary Materials 2).

#### Biological sample collection and test

Peripheral blood (20 ml) and hair samples were collected from participants with their consent before the surgery in accordance with standard sample collection protocols (Supplementary materials 3). Genotyping data are derived from all collected blood samples. In July 2021 and March 2023, we successfully extracted DNA and performed GeneChip sequencing for 1,953 and 3,216 blood samples, respectively. DNA quality control and genotyping were performed at the WeGene Clinical Laboratory, Shenzhen. Genotyping was performed by the Illumina iScan System following the manufacturer’s instructions for the Magen-MD5111 kit. The initial quality control of GeneChip data was processed with Plink2 (pLink®), and we used Eagle2 for haplotype analysis and Minimac4 for genotype imputation, according to the Thousand Genomes Project phase III data.

In addition to GeneChip sequencing, we started a whole-genome sequencing (WGS) program in 2023, which planned to include samples from approximately 3,000 participants (528 samples have been sequenced in January 2023). The case-cohort design is being used as the sample selection strategy. We illustrated the conception of this study design, as well as the process and sample estimates in Supplementary Fig. 1 and Supplementary Table 3. Sequencing data were derived from a DNBSEQ-T7 next-generation sequencer (a target coverage depth of 30×). Genomic variants were called following the modified Genome Analysis Toolkit (GATK) best practice workflow in the Institute of Rare Disease at West China Hospital, Sichuan University [26]. After the standard quality control process, variants were aligned to GRCh38 and annotated using Variant Effect Predictor (VEP), resulting in more than 3 million polymorphic variants remaining for further analysis [27].

### Quality control

**Fig. 2:**
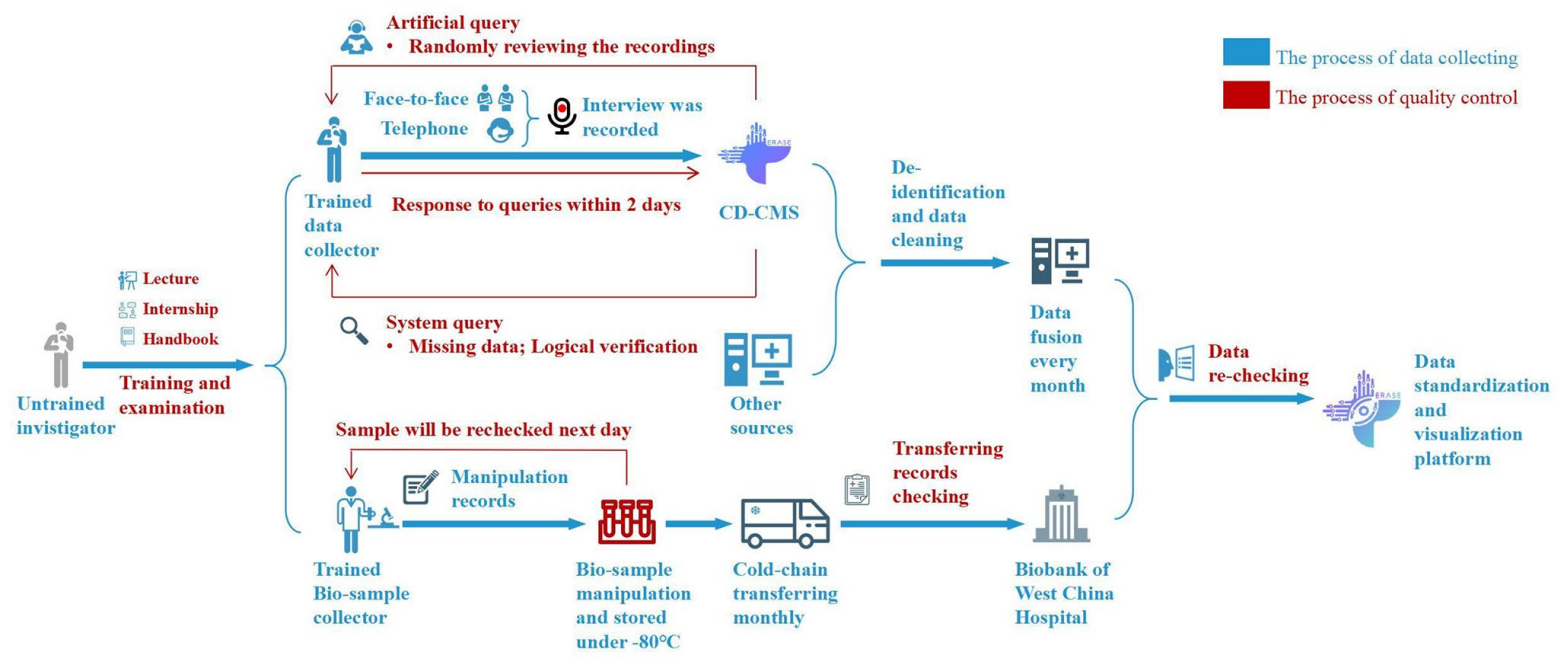
The process of data collection and quality control of the China Surgery and Anesthesia Cohort (CSAC)

Notably, the CSAC was characterized by multiple approaches for stringent quality control (Fig. 2). First, the CSAC has a professional data collection team of 20 full-time investigators, and each data collector and investigator was required to attend a training program (i.e., 14-day lecture and practice courses) and pass the designed examinations before starting their work (Supplementary materials 1). Additionally, we have a multiple-step protocol for assessing the quality of data, including automated logic checks and warnings implemented in CD-CMS, as well as manual audio-recording checks.

### Ethical considerations

All the participants signed separate informed consent for data and biosample collection. The approval for the future use of derived data (including genetic data) extracted from those biosamples has also been specified in the later consent. We take all necessary steps to keep personal information confidential. The study was approved by the ethics committee of West China Hospital, Sichuan University (approval number 2020 − 469). The collection, storage, and use of biosamples were documented and approved by the Ministry of Science and Technology of the People’s Republic of China (approval number 2021-CJ0606).

## Results

By July 18, 2023, a total of 13,484 participants were considered eligible, among whom 12,766 agreed to participate (baseline response rate = 94.68%; flowchart shown in Supplementary Fig. 2). The proportions of participants who agreed to donate blood and hair samples were 69.23% (8,839/12,766) and 70.85% (9,045/12,766), respectively. The follow-up rates at 1 day, 3 days, 7 days, 1 month, 3 months, 6 months and 1 year after surgery were 99.45%, 98.39%, 96.86%, 93.79%, 93.69%, 94.11%, 92.42%, respectively (Supplementary Table 4). The rates for complete follow-ups (i.e., having complete data at all scheduled follow-up points) are shown in Supplementary Table 4.

### Baseline characteristics of the participants

The baseline characteristics of the CSAC are shown in Table 1. In brief, most of these patients belonged to the Han ethnicity (98.78%). The mean age at recruitment was 52.40 years, and there were more females than males (female vs. male = 57.93% vs. 42.07%). Regarding lifestyle factors, 27.29% and 19.18% of the participants were ever smokers and alcohol drinkers, respectively. Approximately 70% of participants had no history of severe diseases (i.e., Charlson comorbidity index [CCI] = 0; the indication disease of the index surgery was not taken into account) before the surgery, and approximately 19% of them experienced chronic pain (lasting more than 1 month). Data on preoperative neuropsychologic conditions revealed that 6.96% of included patients had symptoms of anxiety, 11.11% were suffering from depressive symptoms, 11.02% had cognitive dysfunction manifested as an AD-8 score ≥ 2, and 39.62% had short-term memory impairment (i.e., a three-word recall test score < 3).

**Table 1:** Basic characteristics and surgery/anesthesia-related information of study population

	Overall (*n* = 12,755)	Noncardiac surgery (*n* = 11,808)	Cardiac surgery (*n* = 947)
**Recruitment centers,*n*** (**%)**			
West China Hospital, Sichuan University	10,368 (81.29)	9421 (79.78)	947 (100.00)
West China Tianfu Hospital	1837 (14.40)	1837 (15.56)	0 (0.00)
The First People’s Hospital of Longquanyi District	375 (2.94)	375 (3.18)	0 (0.00)
The Second Hospital of Hebei Medical University	175 (1.37)	175 (1.48)	0 (0.00)
**Tumor diagnosis,*n*** (**%)**			
Yes	6663 (52.24)	6562 (55.57)	101 (10.67)
No	5812 (45.57)	4987 (42.23)	825 (87.12)
Missing	280 (2.2%)	259 (2.2%)	21 (2.2%)
***Social demographic data***			
**Age, years, mean (SD)**	52.40 (6.97)	52.32 (7.01)	53.34 (6.31)
**Sex, n (%)**			
Male	5366 (42.07)	4833 (40.93)	533 (56.28)
Female	7389 (57.93)	6975 (59.07)	414 (43.72)
**Education,*n*** (**%)**			
Middle school and lower	3794 (29.75)	3338 (28.27)	456 (48.15)
High school	2840 (22.27)	2636 (22.32)	204 (21.54)
Junior college	2634 (20.65)	2481 (21.01)	153 (16.16)
College and above	3487 (27.34)	3353 (28.40)	134 (14.15)
**BMI, n (%)**			
< 18.5	452 (3.54)	420 (3.56)	32 (3.38)
18.5–24.9	8621 (67.59)	8033 (68.03)	588 (62.09)
25-29.9	3323 (26.05)	3028 (25.64)	295 (31.15)
≥ 30.0	359 (2.81)	327 (2.77)	32 (3.38)
**Ethnicity,*n*** (**%)**			
Han	12,599 (98.78)	11,686 (98.97)	913 (96.41)
Tibetan	45 (0.35)	36 (0.30)	9 (0.95)
Hui	32 (0.25)	25 (0.21)	7 (0.74)
Others	79 (0.62)	61 (0.52)	18 (1.90)
**Marital status,*n*** (**%)**			
Unmarried	141 (1.11)	137 (1.16)	4 (0.42)
Married	12,045 (94.43)	11,136 (94.31)	909 (95.99)
Divorced	423 (3.32)	404 (3.42)	19 (2.01)
Widowed	146 (1.14)	131 (1.11)	15 (1.58)
**Children situation,*n*** (**%)**			
Living in same city	10,700 (83.89)	10,070 (85.28)	630 (66.53)
Living in another city	1703 (13.35)	1407 (11.92)	296 (31.26)
No children	338 (2.65)	319 (2.70)	19 (2.01)
Children passed away	14 (0.11)	12 (0.10)	2 (0.21)
***Lifestyle factors***			
**Smoking**^**1**^, ***n*(%)**			
Ever	3481 (27.29)	3138 (26.58)	343 (36.22)
Never	9274 (72.71)	8670 (73.42)	604 (63.78)
**Alcohol Drinking**^**2**^, ***n*(%)**			
Ever	2446 (19.18)	2200 (18.63)	246 (25.98)
Never	10,309 (80.82)	9608 (81.37)	701 (74.02)
**Bed time,*n*** (**%)**			
Before 22:00	3951 (30.98)	3551 (30.07)	400 (42.24)
After 22:00	8803 (69.02)	8256 (69.92)	547 (57.76)
Missing	1 (0.0%)	1 (0.0%)	0 (0%)
***Comorbidity***			
**History of psychiatric disorder,*n*** (**%)**			
Yes	249 (1.95)	234 (1.98)	15 (1.58)
No	12,506 (98.05)	11,574 (98.02)	932 (98.42)
**Charlson comorbidity index,*n*** (**%)**			
0	8942 (70.11)	8403 (71.16)	539 (56.92)
1	2562 (20.09)	2274 (19.26)	288 (30.41)
≥ 2	1248 (9.78)	1128 (9.55)	120 (12.67)
Missing	3 (0.02)	3 (0.03)	0 (0.00)
**Preoperative chronic pain**^**3**^, ***n*(%)**			
Yes	2453 (19.23)	2259 (19.13)	194 (20.49)
No	9758 (76.50)	9035 (76.52)	723 (76.35)
Missing	544 (4.3%)	514 (4.4%)	30 (3.2%)
***Preoperative neuropsychologic condition***			
**Symptoms of anxiety (GAD-7 ≥ 5),*n*** (**%)**			
Yes	888 (6.96)	818 (6.93)	70 (7.39)
No	11,864 (93.01)	10,987 (93.05)	877 (92.61)
Missing	3 (0.0%)	3 (0.0%)	0 (0%)
**Symptoms of depression (PHQ-9 ≥ 5),*n*** (**%)**			
Yes	1417 (11.11)	1291 (10.93)	126 (13.31)
No	11,335 (88.87)	10,514 (89.04)	821 (86.69)
Missing	3 (0.0%)	3 (0.0%)	0 (0%)
**Cognitive dysfunction (AD8 ≥ 2),*n*** (**%)**			
Yes	1405 (11.02)	1270 (10.76)	135 (14.26)
No	11,268 (88.34)	10,456 (88.55)	812 (85.74)
Missing	82 (0.6%)	82 (0.7%)	0 (0%)
**Short-term memory impairment (Three-word recall test < 3),** ***n*** (**%)**			
Yes	5053 (39.62)	4633 (39.24)	420 (44.35)
No	7244 (56.79)	6717 (56.89)	527 (55.65)
Missing	458 (3.6%)	458 (3.9%)	0 (0%)
***Anesthesia-related factors***			
**ASA grade,*n*** (**%)**			
I	89 (0.70)	89 (0.75)	0 (0.00)
II	10,509 (82.39)	10,484 (88.79)	25 (2.64)
III	2000 (15.68)	1172 (9.93)	828 (87.43)
IV	97 (0.76)	5 (0.04)	92 (9.71)
V	1 (0.01)	1 (0.01)	0 (0.00)
VI	1 (0.01)	0 (0.00)	1 (0.11)
Missing	58 (0.5%)	57 (0.5%)	1 (0.1%)
**Type of maintenance of general anesthesia, n (%)**			
Combined intravenous and inhalation anesthesia	12,036 (94.36)	11,138 (94.33)	898 (94.83)
Total intravenous anesthesia	538 (4.22)	499 (4.23)	39 (4.12)
Inhalation anesthesia	121 (0.95)	112 (0.95)	9 (0.95)
Missing	60 (0.5%)	59 (0.5%)	1 (0.1%)
**Combined with nerve block**			
Yes	3937 (30.87)	3889 (32.94)	48 (5.07)
No	8818 (69.1%)	7919 (67.1%)	899 (94.9%)
**Anesthesia duration, minute, mean (SD)**	160 (111)	147 (102)	292 (110)
**Severe hypoxia**^**4**^, ***n*(%)**			
Yes	694 (5.44)	522 (4.42)	172 (18.16)
No	8238 (64.59)	7602 (64.38)	636 (67.16)
Missing	3823 (30.0%)	3684 (31.2%)	139 (14.7%)
**Severe hypotension**^**5**^, ***n*(%)**			
Yes	1939 (15.20)	1703 (14.42)	236 (24.92)
No	6914 (54.21)	6342 (53.71)	572 (60.40)
Missing	3902 (30.6%)	3763 (31.9%)	139 (14.7%)
**Patient controlled analgesia after surgery, n (%)**			
Yes	4795 (37.59)	4771 (40.40)	24 (2.53)
No	7902 (61.95)	6980 (59.11)	922 (97.36)
Missing	58 (0.5%)	57 (0.5%)	1 (0.1%)
***Surgery-related factors***			
**Site of surgery,*n*** (**%)**			
Head and neck	2362 (18.52)	2362 (20.00)	0 (0.00)
Thorax	2761 (21.65)	1814 (15.36)	947 (100.00)
Abdomen	4805 (37.67)	4805 (40.69)	0 (0.00)
Limbs and others	2827 (22.16)	2827 (23.94)	0 (0.00)
**Type of surgery,*n*** (**%)**			
Endoscopy	6187 (48.51)	6139 (51.99)	48 (5.07)
Open	6510 (51.04)	5612 (47.53)	898 (94.83)
Missing	58 (0.5%)	57 (0.5%)	1 (0.1%)
**Intraoperative blood transfusion,*n*** (**%)**			
Yes	1181 (9.26)	401 (3.40)	780 (82.37)
No	11,296 (88.56)	11,150 (94.43)	146 (15.42)
Missing	278 (2.2%)	257 (2.2%)	21 (2.2%)
**Admission to ICU after surgery,*n*** (**%)**			
Yes	1146 (8.98)	241 (2.04)	905 (95.56)
No	11,331 (88.84)	11,310 (95.78)	21 (2.22)
Missing	278 (2.2%)	257 (2.2%)	21 (2.2%)
**Length of hospital stay, days, mean (SD)**	8.08 (6.54)	7.74 (6.47)	12.2 (6.01)

^1^ Smoking refers to smoking at least one cigarette every three days for half a year

^2^ Alcohol drinking refers to drinking at least once a week for half a year

^3^ Pain lasts more than 1 month

^4^ Severe hypoxia was defined as pulse oxygen saturation < 90% during the surgery

^5^ Severe hypotension was defined as atrial blood pressure < 60% of baseline level during the surgery

Most of the participants had an American Society of Anaesthesiologists (ASA) grade of II (82.39%) or III (15.68%), and 94.36% of participants received combined intravenous (i.e., propofol) and inhalation (i.e., sevoflurane) anaesthetics for maintenance of general anaesthesia during a mean anaesthesia duration of 160 (standard deviation, SD = 111) mins. During surgery, 5.44%, 15.20%, and 9.26% of patients experienced severe hypoxia (pulse oxygen saturation < 90% during surgery), severe hypotension (atrial blood pressure < 60% of baseline level during surgery) and blood transfusion (autologous or allogeneic transfusion), respectively. The proportions of patients who received surgery of the head and neck, thorax, abdomen, limbs and others were 18.52% (2,362/12,755), 21.65% (2,761/12,755), 37.67% (4,805/12,755) and 22.16% (2,827/12,755), respectively. A total of 6,187 (48.51%) patients underwent endoscopic surgery. The mean length of hospital stay was 8.08 (SD = 6.54) days.

Patients who underwent cardiac surgery were largely different from noncardiac surgery patients in terms of lifestyle factors (e.g., more smokers and alcohol drinkers), comorbidities (e.g., more patients with CCI ≥ 2), preoperative cognitive dysfunction, and surgery/anaesthesia-related factors (e.g., higher ASA grade and longer hospital stay) (Table 1). Nevertheless, the data were largely comparable among participants with and without blood sample collection (Supplementary Table 5).

### Interested surgery/anaesthesia-related outcomes

The incidence rates of surgery and anaesthesia complications are shown in Table 2. Specifically, the most common anaesthesia complication was intraoperative awareness (0.06%), and the top three common surgery complications were pulmonary complications (12.39%), infection (6.61%), and AKI (1.65%). Other anaesthesia- (e.g., failed intubation, aspiration, cardiac arrest) and surgery-related complications (e.g., within-hospital death) were considered relatively rare. Importantly, we generally found higher surgical complication rates among patients who underwent cardiac surgery than among those who underwent noncardiac surgery. For instance, the incidence rate of intraoperative awareness (i.e., if patients regain consciousness under general anaesthesia and are able to recall these events after surgery) was 0.07% among noncardiac surgery patients, and no intraoperative awareness was observed in cardiac surgery patients. Additionally, the incidence of pulmonary complications was 8.88% and 56.18% among participants who received noncardiac and cardiac surgeries, respectively.

**Table 2:** The presence of in-hospital surgery/anesthesia related adversities or complications among study participants

Outcomes, *n* (%)	Overall (*n* = 12,755)	Noncardiac surgery (*n* = 11,808)	Cardiac surgery (*n* = 947)
***During anesthesia***			
Failed intubation	0 (0.00)	0 (0.00)	0 (0.00)
Aspiration	0 (0.00)	0 (0.00)	0 (0.00)
Cardiac arrest	2 (0.02)	2 (0.02)	0 (0.00)
Intraoperative awareness	8 (0.06)	8 (0.07)	0 (0.00)
***After surgery***			
Pulmonary complications^1^	1580 (12.39)	1048 (8.88)	532 (56.18)
Infections^2^	843 (6.61)	697 (5.90)	146 (15.42)
MACE^3^	119 (0.93)	28 (0.24)	91 (9.61)
AKI^4^	211 (1.65)	118 (1.00)	93 (9.82)
Within hospital death	10 (0.08)	7 (0.06)	3 (0.32)

^1^ Pulmonary complications include respiratory infection, respiratory failure, pleural effusion, atelectasis, pneumothorax, bronchospasm and aspiration pneumonia

^2^ Infection include pulmonary infection, surgical site infection, urinary infection and hematogenous infection

^3^ MACE: Major Adverse Cardiovascular Events, include non-fatal cardiac arrest, acute myocardial infarction, congestive heart failure, arrhythmia and angina pectoris

^4^ AKI: Acute kidney injury

Assessments of postoperative pain and neuropsychologic conditions at each follow-up time point are shown in Table 3. Considering the trajectory of studied measurements among patients who received noncardiac surgery, we observed obviously recovering curves (i.e., most severe at the time points adjacent to the index surgery and then became much less severe with longer time of follow) for the incident postoperative pain and a notable deteriorating curve (i.e., incidence gradually increased over the time after surgery) for incident POCD. Nevertheless, the proportion of participants with psychiatric symptoms (including anxiety, depression, stress reaction, and sleep disturbance) was somewhat stable over the surveillance period. Again, the obtained estimates were generally higher among cardiac surgery patients, which however illustrated similar changing trends as those observed among noncardiac surgery patients.

**Table 3:** Measurements of postoperative pain and neuropsychologic conditions for each follow-up period in the study population

	7-days after surgery	1-months after surgery	3 months after surgery	6 months after surgery	12 months after surgery
***Among patients received non-cardiac surgery***					
Number (%) of patients with available data	11,411/11,722 (97.35)	10,706/11,439 (93.59)	9814/10,502 (93.45)	8533/9073 (94.05)	6220/6725 (92.49)
Postoperative pain, *n* (%)	-	3940 (34.44)	1454 (13.84)	779 (8.59)	400 (5.95)
Postoperative cognitive dysfunction (AD8 ≥ 2), *n* (%)	292 (2.49)	934 (8.17)	-	1438 (15.85)	1153 (17.14)
Short-term memory impairment (Three-word recall test < 3), *n* (%)	5357 (45.70)	3993 (34.91)	3360 (31.99)	3543 (39.05)	3587 (53.34)
Anxiety (GAD-7 ≥ 5), *n* (%)	-	559 (4.89)	-	513 (5.65)	309 (4.59)
Depression (PHQ-9 ≥ 5), *n* (%)	-	1399 (12.23)	-	960 (10.58)	703 (10.45)
Stress reaction (PCL-5 ≥ 33), *n* (%)	-	13 (0.11)	-	8 (0.09)	6 (0.09)
Sleep disturbance (PSQI ≥ 16), *n* (%)	-	225 (1.97)	-	143 (1.58)	98 (1.46)
***Among patients received cardiac surgery***					
Number (%) of patients with available data	854/941 (90.75)	887/922 (96.20)	829/858 (96.62)	727/767 (94.78)	587/640 (91.72)
Postoperative pain, *n* (%)	620 (65.89)	-	298 (34.73)	171 (22.29)	89 (13.91)
Postoperative cognitive dysfunction (AD8 ≥ 2), *n* (%)	94 (9.99)	124 (13.45)	-	142 (18.51)	122 (19.06)
Short-term memory impairment (Three-word recall test < 3) *n* (%)	628 (66.74)	526 (57.05)	478 (55.71)	549 (71.58)	408 (63.75)
Anxiety (GAD-7 ≥ 5), *n* (%)	-	60 (6.51)	-	57 (7.43)	42 (6.56)
Depression (PHQ-9 ≥ 5), *n* (%)	-	168 (18.22)	-	93 (12.13)	66 (10.31)
Stress (PCL-5 ≥ 33), *n* (%)	-	3 (0.33)	-	1 (0.13)	4 (0.62)
Sleep disturbance (PSQI ≥ 16), *n* (%)	-	35 (3.80)	-	14 (1.83)	11 (1.72)

Abbreviation: AD-8: the Eight-item Informant Interview to Differentiate Aging and Dementia; GAD-7: Generalized Anxiety Disorder 7-Item Scale; PHQ-9: Patient Health Questionnaire; PCL-5: Posttraumatic Stress Disorder Checklist for DSM-5; PSQI: Pittsburgh Sleep Quality Index

### COVID-19-related information

We retrospectively collected data related to COVID-19 since January 2023, with a response rate of 97.64% (11,823/12,109) up to July 18, 2023. A total of 82.07% (9,703/11,823) of participants reported having COVID-19 infection; 59.26% (5,750/9,703) of the patients confirmed this diagnosis through the positive results of nucleic acid tests or self-tested antigen kits, and the other 39.87% (3,869/9,703) confirmed this diagnosis based on the presence of typical COVID-19 symptoms and suspected contact history. Among infected cases, the most common symptoms were fever (66.97%, 6,498/9,703), cough (57.49%, 5,578/9,703), and muscular soreness (43.82%, 4,252/9,703), and the majority of those core symptoms disappeared within 14 days (74.21%, 7,201/9,703). More than half of infected individuals (74.44%, 7,223/9,703) took medication (e.g., ibuprofen, acetaminophen) for symptom release without medical care system contacts, 10.10% (980/9,703) received primary or outpatient care, and merely 1.68% (163/9,703) were admitted to a hospital for inpatient care (Supplementary Table 6).

## Discussion

### CSAC in comparison with other studies

No comparable data from cohorts with identical study designs (i.e., with similar study populations, inclusion sites, and sample sizes) could be found. One relevant large international cohort (VISION) recruited noncardiac surgery patients with a focus on certain selected outcomes (i.e., vascular events) [28]. However, due to demographic differences (e.g., population and age group) and heterogeneities in study settings (e.g., distribution of indication diseases), the basic characteristics of our study population were largely different from those in the VISION cohort. Investigations focusing on middle-aged Chinese surgery patients are also scarce. Regarding the incidence rates of postoperative complications, we observed that the incidence of postoperative pulmonary complications after noncardiac surgery in the CSAC was 8.88%, which was similar to the rate of 7.6% reported in a previous study [29]. We observed a lower rate for postoperative infections than a previous study (5.90% vs. 9.8% in the VISION cohort [30]). Again, given the enormous variations in many aspects (e.g., age and the percentage of endoscopic surgery), the differences in findings between those studies did not necessarily invalidate each other.

### Key findings to date and further study plan

Based on the assessments of postoperative pain and neuropsychologic conditions at each follow-up time point, we conducted studies analysing risk factors associated with the occurrence of CPSP (i.e., pain in the surgical area persisted for more than 3 months after surgery) [31] and POCD (i.e., indexed by AD-8 score ≥ 2) [32]. Our results indicated that preoperative psychopathology, particularly the presence of anxiety, depression, and sleep disturbance, was a significant risk factor for the occurrence of CPSP and POCD. Furthermore, some anaesthesia- and surgery-related factors, including a longer duration of general anaesthesia (e.g., > 180 min) and postoperative complications (e.g., postoperative pulmonary complications, acute kidney injury), were also linked to the studied adversities (Supplementary Table 7). These findings derive from our initial efforts to decipher the notable postoperative adversities, and the results have the potential to prompt further mechanistic exploration and facilitate the development of effective approaches for CPSP and POCD prevention among middle-aged Chinese individuals. Subsequent studies, such as genome-wide association analyses (GWAS) for identifying genetic determinants of CPSP and POCD, are ongoing.

### Strengths and weaknesses

To the best of our knowledge, this is the first cohort involving a large number of patients who underwent surgery and general anaesthesia in China. In addition to the professional data collection team and stringent quality control process, which ensure the accuracy and completeness of our data (baseline response rate 94.68% and 92.42%-99.45% follow-up rates within 1 year after surgery), the CSAC was most characterized by the potential of providing multidimensional data, including biological information extracted from biosamples, and advancing the current understanding of the joint effects of medical, environmental, psychological, and genetic factors on the prognosis of surgery patients. We have further built an integrated platform to realize automate linkages between different data resources (every 3 months) and facilitate remote data access to the centralized database (after deidentification).

There are also notable limitations. Due to the late involvement of medical centres outside Sichuan Province, this cohort currently has poor representativeness of all surgery patients among the Chinese population. However, as the study is still ongoing and we have planned to include more recruitment centres in other locations, the geographical distribution of the study participants will gradually change. Second, as we only collected biosamples at baseline, research on adynamic biomarkers for prognosis prediction or evaluations of the effectiveness of some medical interventions will not be possible. Third, although most of the used scales have been widely validated in community-based samples, including the Chinese population [16–21, 23], the lack of more professional assessments on some interested consequences, such as medical diagnosis of psychiatric disorders or cognitive impairment, will make it more difficult to measure these outcomes or other outcomes without clinical significance. In addition, participants in the cardiac surgery cohort were invited to join this cohort midway due to practical reasons, and they are likely to have different perioperative conditions. We therefore encourage separate analyses for cardiac and noncardiac surgery patients. Last, it is notable that the nature of the patient cohort design limits the possibility of studying consequences related to the presence or absence of general anaesthesia during surgery (which requires comparisons between individuals with and without general anaesthesia during surgery). However, as in most cases, receiving surgery is basically inevitable for curing a disease, and such a patient cohort is also useful in terms of improving the prognosis of surgery patients by clarifying the approaches that can optimize the perioperative procedure.

### Collaboration

Currently, the data of CSAC are not available to public for data protection and privacy policy. But we welcome clinicians and researchers contact us, for initiating collaborative projects or joining CSAC as a new recruitment center. Please visit our website at http://biomedbdc.wchscu.cn/JoylabErasePM/hx-portals/#/index for more details, and contact corresponding authors Huan Song (songhuan@wchscu.cn) or Qian Li (hxliqian@wchscu.cn) for collaboration requests.

### Electronic supplementary material

Below is the link to the electronic supplementary material.

Supplementary Material 1
