# Supplementary material for: Cohort profile: the China surgery and anesthesia cohort (CSAC)
Source: Eur J Epidemiol. 2024 Jan 10;39(2):207–18. doi: 10.1007/s10654-023-01083-4 (PMC10904502; doi:10.1007/s10654-023-01083-4)
Supplement: Supplementary file 1 — Supplementary Material 1 [file 10654_2023_1083_MOESM1_ESM.docx]

**Supplementary**

**Supplementary materials 1** Investigator Training Course Checklist

**Supplementary materials 2** Covid-19 history collection form

**Supplementary materials 3** Bio-sample collection protocols

**Supplementary Figure 1** Case-cohort design for Genome sequencing program

**Supplementary Figure 2** Study flowchart

**Supplementary Table 1** Measurements of collected data in the CSAC

**Supplementary Table 2** The cut-off points of neuropsychological assessment scales

**Supplementary Table 3** Estimated sample size for Sequencing participants using the case-cohort design

**Supplementary Table 4** The follow-up rate at each time point

**Supplementary Table 5** Basic characteristics and surgery/anesthesia-related information of study population by bio-sample collection

**Supplementary Table 6** Data relevant to the COVID-19 of the CSAC

**Supplementary Table 7** Risk factors for the occurrence of CPSP and POCD among non-cardiac surgical patients

**Supplementary materials 1** Investigator Training Course Checklist

1. Preparation

1.1 Introduce the hospital environment and members of the project team. □

1.2 Introduce the objective, design and implementation of the study. □

1.3 Introduce the rules and regulations of the project team. □

1.4 Share relative materials, such as investigator handbook, tablet PC, office supplies et.al. □

2. Training course: Details of the Investigator handbook

2.1 Screening process:

□ Pass □ Reject Trainer Signature_____________

2.2 Informed consent:

□ Pass □ Reject Trainer Signature_____________

2.3 Data collection at baseline:

□ Pass □ Reject Trainer Signature_____________

2.4 Data collection at follow up period:

□ Pass □ Reject Trainer Signature_____________

2.5 Communication skills:

□ Pass □ Reject Trainer Signature_____________

**Supplementary materials 2** Covid-19 history collection form

1. What kind of COVID-19 vaccine have you received：

□ not vaccinated

□ vaccinated（multiple choice：□ Kexing □ Sinopharm □ Other______ □ Not sure）

2. You have received ____ time(s) vaccination, last vaccination time ___year___month ___day

3. You have been infected with Covid-19 ____ time(s)

3.1. Diagnosis method for Covid-19 infection（multiple choice）

□ nucleic acid testing diagnosis time：___year___month ___day

□ antigen testing diagnosis time：___year___month ___day

□ typical symptom without testing

□ no typical symptom

3.2.Onset time of symptoms of Covid-19 infection ：___year___month ___day

3.3.Persist time of symptoms of Covid-19 infection：

□ 1-2 day □ 3-5 days □ 5-8 days □ 8-14 days □ >14 days □ during infection, ___day

3.4.symptoms during infection：

□ fever

highest temperature：□ >39.5℃ □ 38.6-39.5℃ □ 37.5-38.5℃；

persist time：□ 1-2 day □ 3-5 days □ 5-8 days □ 8-14 days □ >14 days □d uring infection, ___day

□ shivering

persist time：□ 1-2 day □ 3-5 days □ 5-8 days □ 8-14 days □ >14 days □ during infection, ___day

□ sniffling or stuffiness

persist time：□ 1-2 day □ 3-5 days □ 5-8 days □ 8-14 days □ >14 days □during infection, ___day

□ cough

persist time：□ 1-2 day □3-5 days □ 5-8 days □ 8-14 days □ >14 days □ during infection, ___day

□ sore throat

persist time：□ 1-2 day □ 3-5 days □ 5-8 days □ 8-14 days □ >14 days □ during infection, ___day

□ tachypnea or dyspnea

multiple choice：□ dyspnea after light activity □ nocturnal paroxysmal dyspnea □orthopnea □continuous oxygen；

persist time：□ 1-2 day □ 3-5 days □ 5-8 days □ 8-14 days □ >14 days □ during infection, ___day

□ fatigue

persist time：□ 1-2 day □ 3-5 days □ 5-8 days □ 8-14 days □ >14 days □ during infection, ___day

□ headache

persist time：□ 1-2 day □ 3-5 days □ 5-8 days □ 8-14 days □ >14 days □ during infection, ___day

□ Muscular soreness

multiple choice：□ limbs pain □ trunk pain

persist time：□ 1-2 day □ 3-5 days □ 5-8 days □ 8-14 days □ >14 days □ during infection, ___day

□ Hypogeusia

multiple choice：□ sour □ sweet □ bitter □ salty；

persist time：□ 1-2 day □ 3-5 days □ 5-8 days □ 8-14 days □ >14 days □ during infection, ___day

□ Hyposmia

persist time：□ 1-2 day □3-5 days □ 5-8 days □ 8-14 days □ >14 days □during infection, ___day

□ nausea and vomiting

persist time：□ 1-2 day □ 3-5 days □ 5-8 days □ 8-14 days □ >14 days □during infection, ___day

□ diarrhea

persist time：□ 1-2 day □ 3-5 days □ 5-8 days □ 8-14 days □ >14 days □ during infection, ___day

3.5 Treatment received：

□ Medicine for alleviating symptoms（such as ibuprofen, acetaminophen）

□ Traditional Chinese medicine（lianhua qingwen）

□ Oxygen at home：oxygen flow rate ___L/min，Oxygen saturation after oxygen__%，□Oxygen saturation was not measured

□ Hospitalization：□ Continuous oxygenation □ Non-invasive ventilation □ Invasive ventilation □ ECMO

□ Not received any treatment

□ Others (such as take antiviral drugs or received intravenous or intramuscular injection in clinic)，please clarify：

**Supplementary materials 3** Bio-sample collection protocols

- Blood samples: Peripheral blood (20 ml) was drawn from each patient. Ten milliliters of blood were collected into ethylenediamine tetraacetic acid (EDTA) tubes for separating plasma and cells. The blood was immediately stored in a refrigerator at 4℃ after collection. The other 10 ml blood was collected into clot activator serum separation tubes (SSTs) for separating serum. The blood was kept at room temperature for 30-45 minutes and then stored in a refrigerator at 4°C. All blood samples were centrifuged within 4 hours after collection at 3000 r* 15 min*4°C. After centrifugation, plasma, cells and serum were separately sucked into cryogenic vials and stored in a refrigerator at -80°C.
- Hair samples: At 5 cm below the patient’s occipital tuberosity, 3 cm hair from the root was cut off. Hair samples are kept in specimen bags with identified code.

From the full CASC cohort, select a sub-cohort of individuals at baseline

(The sub-cohort will include some cases)

Identify specific cases that occur outside the sub-cohort during follow-up

Perform WGS for sub-cohort and cases outside sub-cohort

Same sub-cohort can be used for several diseases (cases/outcome)

**Sub-cohort (random selected)**

**Cases (cases 1, case 2, cases 3... )**

**CASC cohort**

**Supplementary Figure 1** Case-cohort design for Genome sequencing program

**Supplementary Figure 2** Study flowchart (updated on 2023.July.18)

12,755 included in the follow-up visit

8,839 patients donated blood samples

9,045 patients donated hair

13,484 participants were eligible

Excluded

718 refused to participant

Excluded

11 withdraw their consent

12,766 agreed to participate

**Supplementary Table 1** Measurements of collected data in the CSAC

| Variables | Measurements | Data sources | Baseline | Follow-up | | | | | | |
| --- | --- | --- | --- | --- | --- | --- | --- | --- | --- | --- |
|  |  |  |  | POD1 | POD3 | POD7 | POM1 | POM3 | POM6 | POY1 |
| **Sociodemographic data** | | | | | | | | | | |
| Age, sex, education, BMI, Ethnicity, marital status, children situation | - | Face-to-face interview | √ |  |  |  |  |  |  |  |
| **Life style** |  |  |  |  |  |  |  |  |  |  |
| Smoking, Drinking | - | Face-to-face interview | √ |  |  |  |  |  |  |  |
| **Physical condition** |  |  |  |  |  |  |  |  |  |  |
| Comorbidity | Charlson index | Face-to-face interview | √ |  |  |  |  |  |  |  |
| Pain | BPI | Face-to-face or telephone interview | √ | √ | √ |  | √ | √ | √ | √ |
| **Psychological condition** | | | | | | | | | | |
| Sleep quality | PSQI | Face-to-face or telephone interview | √ |  |  |  | √ |  | √ | √ |
| Stress | IES-R or PCL-5 | Face-to-face or telephone interview | √ |  |  |  | √ |  | √ | √ |
| Depression | PHQ-9 | Face-to-face or telephone interview | √ |  |  |  | √ |  | √ | √ |
| Anxiety | GAD-7 | Face-to-face or telephone interview | √ |  |  |  | √ |  | √ | √ |
| **Cognitive condition** | | | | | | | | | | |
| Subjective cognitive function | AD8 | Face-to-face or telephone interview | √ |  |  | √ | √ |  | √ | √ |
| Short-term memory | Three word recall test | Face-to-face or telephone interview | √ |  |  | √ | √ |  | √ | √ |
| **Complications** | | | | | | | | | | |
| Anesthesia-related：postoperative nausea and vomiting, severe hypoxia, aspiration, cardiac arrest, intraoperative awareness | EPCO definitions | Medical record |  | √ | √ |  |  |  |  |  |
| Surgical-related：myocardial infarction, pulmonary embolism (PE), stroke, acute kidney injury (AKI), acute respiratory distress syndrome (ARDS), anastomotic breakdown, postoperative infection, cardiac arrest | EPCO definitions | Medical record |  |  |  | √ |  |  |  |  |
| Death |  | Medical record, telephone interview, or linked administrative data |  | √ | √ | √ | √ | √ | √ | √ |
| **Sample collection** | | | | | | | | | | |
| Blood and hair samples |  | - | √ |  |  |  |  |  |  |  |

Abbreviation: POD: postoperative day; POM: postoperative month; POY: postoperative year; BPI: Brief Pain Inventory; PSQI: Pittsburgh Sleep Quality Index; IES-R: Impact of Event Scale-Revised; PCL-5: Post-traumatic Stress Disorder Checklist for DSM-5; PHQ-9: Patient Health Questionnaire; GAD-7: Generalized Anxiety Disorder 7-item scale; AD-8: Eight-item Informant Interview to Differentiate Aging and Dementia; EPCO: European Perioperative Clinical Outcome.

**Supplementary Table 2** The cut-off points of neuropsychological assessment scales

| **Scales** | **Cut-off points** | **References** |
| --- | --- | --- |
| Pittsburgh Sleep Quality Index [PSQI] | > 6 | Zheng B, Li M, Wang KL, Lv J. [Analysis of the reliability and validity of the Chinese version of Pittsburgh sleep quality index among medical college students]. Beijing Da Xue Xue Bao Yi Xue Ban. 2016 Jun 18;48(3):424-8. Chinese. PMID: 27318902. |
| Impact of Event Scale-Revised version [IES-R] | ≥ 35 | Guoping H, Yalin Z, hui X, et al. The Chinese Version of The impact of event scale-revised of male offenders: Reliability and validity. Chin J Ment Health. 2006;20:28–31. |
| Posttraumatic Stress Disorder Checklist for DSM-5 [PCL-5] | > 37 | Jiang C, Xue G, Yao S, Zhang X, Chen W, Cheng K, Zhang Y, Li Z, Zhao G, Zheng X, Bai H. Psychometric properties of the post-traumatic stress disorder checklist for DSM-5 (PCL-5) in Chinese stroke patients. BMC Psychiatry. 2023 Jan 9;23(1):16. doi: 10.1186/s12888-022-04493-y. PMID: 36624414 |
| Generalized Anxiety Disorder 7-Item Scale [GAD-7] | ≥ 5 | Zhang C, Wang T, Zeng P, et al. Reliability, Validity, and Measurement Invariance of the General Anxiety Disorder Scale Among Chinese Medical University Students. Frontiers in psychiatry. 2021;12:648755. doi:10.3389/fpsyt.2021.648755 |
| Patient Health Questionnaire [PHQ-9] | ≥ 7 | Wang W, Bian Q, Zhao Y, et al. Reliability and validity of the Chinese version of the Patient Health Questionnaire (PHQ-9) in the general population. General hospital psychiatry. Sep-Oct 2014;36(5):539-44. doi:10.1016/j.genhosppsych.2014.05.021 |
| Eight-item Informant Interview to Differentiate Aging and Dementia [AD-8] | ≥ 2 | Li T, Wang HL, Yang YH, Galvin JE, Morris JC, Yu X. [The reliability and validity of Chinese version of AD8]. Zhonghua Nei Ke Za Zhi. 2012 Oct;51(10):777-80. Chinese. PMID: 23290975. |
| Three-word recall test | **< 3** | Li X, Dai J, Zhao S, Liu W, Li H. Comparison of the value of Mini-Cog and MMSE screening in the rapid identification of Chinese outpatients with mild cognitive impairment. Medicine (Baltimore). 2018 Jun;97(22):e10966. doi: 10.1097/MD.0000000000010966. PMID: 29851846 |

**Supplementary Table 3** Estimated sample size for Sequencing participants using the case-cohort design

|  | Number of participants |
| --- | --- |
| Target total participants with blood sample in the CSAC | ~15,000 |
| Sub-cohort (10%) | ~1,500 |
| Cases of interested outcomes outside the sub-cohort | About 1500 |
| Severe psychiatric symptoms (incidence of 4%) | 540 |
| Severe surgery/anesthesia complications (incidence of 2%) | 270 |
| Postoperative cognitive dysfunction (incidence of 3%) | 405 |
| Postoperative chronic pain (incidence of 2%) | 270 |
| Number of needed subjects. | About 3000 |

**Supplementary Table 4** The follow-up rate at each time point

| **Time after surgery** | **Follow-up rate1** | **Follow-up rate 2** |
| --- | --- | --- |
| 1 day | 12658/12728(99.45%) | 12658/12728(99.45%) |
| 3 days | 12505/12709 (98.39%) | 12468/12709 (98.10%) |
| 7 days | 12265/12663 (96.86%) | 12115/12663 (95.67%) |
| 1 month | 11593/12361 (93.79%) | 11204/12361 (90.64%) |
| 3 months | 10643/11360 (93.69%) | 9779/11360 (86.08%) |
| 6 months | 9260/9840 (94.11%) | 8256/9840 (83.90%) |
| 12 months | 6807/7365 (92.42%) | 5865/7365 (79.63%) |

Follow-up rate1: Rate of participants with complete data at the specific follow-up time point.

Follow-up rate2: Rate of participants with complete data at all time points before the specific follow-up time point.

**Supplementary Table 5** Basic characteristics and surgery/anesthesia-related information of study population by bio-sample collection

|  | Overall (n=12755) | With blood sample collected (n=8839) | Without blood sample collected (n=3916) |
| --- | --- | --- | --- |
| **Recruitment centers, n (%)** |  |  |  |
| West China Hospital, Sichuan University | 10368 (81.29) | 7702 (87.14) | 2666 (68.08) |
| West China Tianfu Hospital, Sichuan University | 1837 (14.40) | 1137 (12.86) | 700 (17.88) |
| The First People's Hospital of Longquanyi District | 375 (2.94) | 0 (0.00) | 375 (9.58) |
| The Second Hospital of Hebei Medical University | 175 (1.37) | 0 (0.00) | 175 (4.47) |
| **Tumor diagnosis, n (%)** |  |  |  |
| Yes | 6663 (52.24) | 4725 (53.46) | 1938 (49.49) |
| No | 5812 (45.57) | 3957 (44.77) | 1855 (47.37) |
| Missing | 280 (2.2%) | 157 (1.8%) | 123 (3.1%) |
| ***Social demographic data*** |  |  |  |
| **Age, years, mean (SD)** | 52.40 (6.97) | 52.34 (7.01) | 52.53 (6.86) |
| **Sex, n (%)** |  |  |  |
| Male | 5366 (42.07) | 3774 (42.70) | 1592 (40.65) |
| Female | 7389 (57.93) | 5065 (57.30) | 2324 (59.35) |
| **Education, n (%)** |  |  |  |
| Middle school and lower | 3794 (29.75) | 2462 (27.85) | 1332 (34.01) |
| High school | 2840 (22.27) | 1970 (22.29) | 870 (22.22) |
| Junior college | 2634 (20.65) | 1885 (21.33) | 749 (19.13) |
| College and above | 3487 (27.34) | 2522 (28.53) | 965 (24.64) |
| **BMI, n (%)** |  |  |  |
| <18.5 | 452 (3.54) | 316 (3.58) | 136 (3.47) |
| 18.5-24.9 | 8621 (67.59) | 6056 (68.51) | 2565 (65.50) |
| 25-29.9 | 3323 (26.05) | 2254 (25.50) | 1069 (27.30) |
| ≥ 30.0 | 359 (2.81) | 213 (2.41) | 146 (3.73) |
| **Ethnicity, n (%)** |  |  |  |
| Han | 12599 (98.78) | 8732 (98.79) | 3867 (98.75) |
| Tibetan | 45 (0.35) | 32 (0.36) | 13 (0.33) |
| Hui | 32 (0.25) | 21 (0.24) | 11 (0.28) |
| Others | 79 (0.62) | 54 (0.61) | 25 (0.64) |
| **Marital status, n (%)** |  |  |  |
| Unmarried | 141 (1.11) | 97 (1.10) | 44 (1.12) |
| Married | 12045 (94.43) | 8387 (94.89) | 3658 (93.41) |
| Divorced | 423 (3.32) | 267 (3.02) | 156 (3.98) |
| Widowed | 146 (1.14) | 88 (1.00) | 58 (1.48) |
| **Children situation, n (%)** |  |  |  |
| Living in same city | 10700 (83.89) | 7396 (83.67) | 3304 (84.37) |
| Living in another city | 1703 (13.35) | 1204 (13.62) | 499 (12.74) |
| No children | 338 (2.65) | 231 (2.61) | 107 (2.73) |
| Children passed away | 14 (0.11) | 8 (0.09) | 6 (0.15) |
| ***Lifestyle factors*** |  |  |  |
| **Smoking^1^, n (%)** |  |  |  |
| Ever | 3481 (27.29) | 2434 (27.54) | 1047 (26.74) |
| Never | 9274 (72.71) | 6405 (72.46) | 2869 (73.26) |
| **Alcohol Drinking^2^, n (%)** |  |  |  |
| Ever | 2446 (19.18) | 1698 (19.21) | 748 (19.10) |
| Never | 10309 (80.82) | 7141 (80.79) | 3168 (80.90) |
| **Bed time, n (%)** |  |  |  |
| Before 22:00 | 3951 (30.98) | 2643 (29.90) | 1308 (33.40) |
| After 22:00 | 8803 (69.02) | 6196 (70.10) | 2607 (66.57) |
| Missing | 1 (0.0%) | 0 (0%) | 1 (0.0%) |
| ***Comorbidity*** |  |  |  |
| **History of psychiatric disorder, n (%)** |  |  |  |
| Yes | 249 (1.95) | 164 (1.86) | 85 (2.17) |
| No | 12506 (98.05) | 8675 (98.14) | 3831 (97.83) |
| **Charlson comorbidity index, n (%)** |  |  |  |
| 0 | 8942 (70.11) | 6293 (71.20) | 2649 (67.65) |
| 1 | 2562 (20.09) | 1809 (20.47) | 753 (19.23) |
| ≥ 2 | 1248 (9.78) | 735 (8.32) | 513 (13.10) |
| Missing | 3 (0.02) | 2 (0.02) | 1 (0.03) |
| **Preoperative chronic pain^3^, n (%)** |  |  |  |
| Yes | 2453 (19.23) | 1725 (19.52) | 728 (18.59) |
| No | 9758 (76.50) | 6971 (78.87) | 2787 (71.17) |
| Missing | 544 (4.3%) | 143 (1.6%) | 401 (10.2%) |
| ***Preoperative neuropsychologic condition*** |  |  |  |
| **Symptoms of anxiety (GAD-7 ≥ 5), n (%)** |  |  |  |
| Yes | 888 (6.96) | 597 (6.75) | 291 (7.43) |
| No | 11864 (93.01) | 8242 (93.25) | 3622 (92.49) |
| Missing | 3 (0.0%) | 0 (0%) | 3 (0.1%) |
| **Symptoms of depression (PHQ-9 ≥ 5), n (%)** |  |  |  |
| Yes | 1417 (11.11) | 918 (10.39) | 499 (12.74) |
| No | 11335 (88.87) | 7920 (89.60) | 3415 (87.21) |
| Missing | 3 (0.0%) | 1 (0.0%) | 2 (0.1%) |
| **Cognitive dysfunction (AD8 ≥ 2), n (%)** |  |  |  |
| Yes | 1405 (11.02) | 920 (10.41) | 485 (12.39) |
| No | 11268 (88.34) | 7884 (89.20) | 3384 (86.41) |
| Missing | 82 (0.6%) | 35 (0.4%) | 47 (1.2%) |
| **Short-term memory impairment (Three-word recall test < 3) n (%)** |  |  |  |
| Yes | 5053 (39.62) | 3515 (39.77) | 1538 (39.27) |
| No | 7244 (56.79) | 5228 (59.15) | 2016 (51.48) |
| Missing | 458 (3.6%) | 96 (1.1%) | 362 (9.2%) |
| ***Anesthesia-related factors*** |  |  |  |
| **ASA grade, n (%)** |  |  |  |
| Ⅰ | 89 (0.70) | 51 (0.58) | 38 (0.97) |
| Ⅱ | 10509 (82.39) | 7252 (82.05) | 3257 (83.17) |
| Ⅲ | 2000 (15.68) | 1446 (16.36) | 554 (14.15) |
| Ⅳ | 97 (0.76) | 85 (0.96) | 12 (0.31) |
| Ⅴ | 1 (0.01) | 1 (0.01) | 0 (0.00) |
| Ⅵ | 1 (0.01) | 1 (0.01) | 0 (0.00) |
| Missing | 58 (0.5%) | 3 (0.0%) | 55 (1.4%) |
| **Type of maintenance of general anesthesia, (%)** |  |  |  |
| Combined intravenous and inhalation anesthesia | 12036 (94.36) | 8357 (94.55) | 3679 (93.95) |
| Total intravenous anesthesia | 538 (4.22) | 406 (4.59) | 132 (3.37) |
| Inhalation anesthesia | 121 (0.95) | 72 (0.81) | 49 (1.25) |
| Missing | 60 (0.5%) | 4 (0.0%) | 56 (1.4%) |
| **Combined with nerve block, (%)** |  |  |  |
| Yes | 3937 (30.87) | 2808 (31.77) | 1129 (28.83) |
| No | 8818 (69.1%) | 6031 (68.2%) | 2787 (71.2%) |
| **Anesthesia duration, minute, mean (SD)** | 160 (111) | 162 (102) | 151 (136) |
| **Severe hypoxia^4^, n (%)** |  |  |  |
| Yes | 694 (5.44) | 533 (6.03) | 161 (4.11) |
| No | 8238 (64.59) | 6285 (71.11) | 1953 (49.87) |
| Missing | 3823 (30.0%) | 2021 (22.9%) | 1802 (46.0%) |
| **Severe hypotension^5^, n (%)** |  |  |  |
| Yes | 1939 (15.20) | 1435 (16.23) | 504 (12.87) |
| No | 6914 (54.21) | 5325 (60.24) | 1589 (40.58) |
| Missing | 3902 (30.6%) | 2079 (23.5%) | 1823 (46.6%) |
| **Patient controlled analgesia after surgery, (%)** |  |  |  |
| Yes | 4795 (37.59) | 3262 (36.90) | 1533 (39.15) |
| No | 7902 (61.95) | 5574 (63.06) | 2328 (59.45) |
| Missing | 58 (0.5%) | 3 (0.0%) | 55 (1.4%) |
| ***Surgery-related factors*** |  |  |  |
| **Site of surgery, n (%)** |  |  |  |
| Head and neck | 2362 (18.52) | 1881 (21.28) | 481 (12.28) |
| Thorax | 2761 (21.65) | 2301 (26.03) | 460 (11.75) |
| Abdomen | 4805 (37.67) | 3165 (35.81) | 1640 (41.88) |
| Limbs and others | 2827 (22.16) | 1492 (16.88) | 1335 (34.09) |
| **Type of surgery, n (%)** |  |  |  |
| Endoscopy | 6187 (48.51) | 4249 (48.07) | 1938 (49.49) |
| Open | 6510 (51.04) | 4587 (51.90) | 1923 (49.11) |
| Missing | 58 (0.5%) | 3 (0.0%) | 55 (1.4%) |
| **Intraoperative blood transfusion, n (%)** |  |  |  |
| Yes | 1181 (9.26) | 979 (11.08) | 202 (5.16) |
| No | 11296 (88.56) | 7703 (87.15) | 3593 (91.75) |
| Missing | 278 (2.2%) | 157 (1.8%) | 121 (3.1%) |
| **Admission to ICU after surgery, n (%)** |  |  |  |
| Yes | 1146 (8.98) | 976 (11.04) | 170 (4.34) |
| No | 11331 (88.84) | 7706 (87.18) | 3625 (92.57) |
| Missing | 278 (2.2%) | 157 (1.8%) | 121 (3.1%) |
| **Length of hospital stay, days, mean (SD)** | 8.08 (6.54) | 7.99 (6.81) | 8.28 (5.88) |

^1^ Smoking refers to smoking at least one cigarette every three days for half a year in recent one year.

^2^ Alcohol drinking refers to drinking at least once a week for half a year.

^3^ Pain lasts more than 1 month.

^4^ Severe hypoxia was defined as pulse oxygen saturation < 90% during the surgery.

^5^ Severe hypotension was defined as atrial blood pressure < 60% of baseline level during the surgery

**Supplementary Table 6** Data relevant to the COVID-19 of the CSAC

| Total participants | Overall | Non-cardiac Surgery | Cardiac surgery |
| --- | --- | --- | --- |
|  | n=11823 | n=10944 | n=879 |
| Have received at least one vaccination (%) | 10126 (85.65%) | 9357 (85.50%) | 769 (87.49%) |
| Have been infected with Covid-19 (%) | 9703 (82.07%) | 8824 (80.63%) | 879 (100.00%) |
| Total infected participants | Overall | Non-cardiac Surgery | Cardiac surgery |
|  | n=9703 | n=8824 | n=879 |
| Diagnosed with nucleic acid testing | 1243 (12.81%) | 1128 (12.78%) | 115 (13.08%) |
| Diagnosed with antigen testing | 4507 (46.45%) | 4317 (48.92%) | 190 (21.62%) |
| Typical symptom without testing | 3869 (39.87%) | 3506 (39.73%) | 363 (41.30%) |
| Symptoms |  |  |  |
| Asymptomatic (%) | 303 (3.12%) | 284 (3.22%) | 19 (2.16%) |
| Fever (%) | 6498 (66.97%) | 6085 (68.96%) | 413 (46.99%) |
| Shivering (%) | 1884 (19.42%) | 1683 (19.07%) | 201 (22.87%) |
| Cough (%) | 5578 (57.49%) | 5165 (58.53%) | 413 (46.99%) |
| Sore throat (%) | 3419 (35.24%) | 3166 (35.88%) | 253 (28.78%) |
| Diarrhea (%) | 683 (7.04%) | 631 (7.15%) | 52 (5.92%) |
| Nausea and vomiting (%) | 522 (5.38%) | 472 (5.35%) | 50 (5.69%) |
| Hyposmia (%) | 1432 (14.76%) | 1331 (15.08%) | 101 (11.49%) |
| Hypogeusia (%) | 2238 (23.07%) | 2079 (23.56%) | 159 (18.09%) |
| Muscular soreness (%) | 4252 (43.82%) | 3956 (44.83%) | 296 (33.67%) |
| Headache (%) | 2301 (23.71%) | 2118 (24.00%) | 183 (20.82%) |
| Fatigue (%) | 3482 (35.89%) | 3195 (36.21%) | 287 (32.65%) |
| Tachypnea or dyspnea (%) | 683 (7.04%) | 605 (6.86%) | 78 (8.87%) |
| sniffling or stuffiness (%) | 1793 (18.48%) | 1645 (18.64%) | 148 (16.84%) |
| Symptom persist time (%) |  |  |  |
| 1-2 days | 1048 (10.80%) | 969 (10.98%) | 79 (8.99%) |
| 3-5 days | 1965 (20.25%) | 1786 (20.24%) | 179 (20.36%) |
| 5-8 days | 2670 (27.52%) | 2467 (27.96%) | 203 (23.09%) |
| 8-14 days | 1518 (15.64%) | 1437 (16.29%) | 81 (9.22%) |
| >14 days | 1866 (19.23%) | 1774 (20.10%) | 92 (10.47%) |
| during infection | 70 (0.72%) | 69 (0.78%) | 1 (0.11%) |
| missing | 566 (5.80%) | 322 (3.6%) | 244 (27.80%) |
| Treatment |  |  |  |
| Not received any treatment (%) | 1404 (14.47%) | 1310 (14.85%) | 94 (10.69%) |
| Take medicine without medical care system contacts (such as ibuprofen, acetaminophen) (%) | 7223 (74.44%) | 6756 (76.56%) | 467 (53.13%) |
| Received primary or outpatient care (%) | 980 (10.10%) | 875 (9.92%) | 105 (11.95%) |
| Hospitalization (%) | 163 (1.68%) | 147 (1.67%) | 16 (1.82%) |
| Non-pharmacotherapy | 69 (0.71%) | 66 (0.75%) | 3 (0.34%) |

**Supplementary Table 7** Risk factors for the occurrence of CPSP and POCD among non-cardiac surgical patients.

| Potential risk factors | Risk of CPSP | Risk of subjective cognitive impairment |
| --- | --- | --- |
|  | OR (95% CI)^1^ | OR (95% CI)^1^ |
| **Psychologic condition** |  |  |
| Anxiety (GAD-7 ≥ 5 vs <5) | 2.16 (1.45-3.24) | 2.55 (2.09-3.12) |
| Depression (PHQ-9 ≥ 5 vs <5) | 0.81 (0.57-1.16) | 2.43 (2.07-2.86) |
| Sleep disturbance (PSQI) |  |  |
| 0~5 | Ref | Ref |
| 6~10 | 1.48 (1.17-1.88) | 1.60 (1.60-1.60) |
| 11~15 | 2.02 (1.41-2.88) | 2.84 (2.84-2.84) |
| ≥ 16 | 1.11 (0.38-3.18) | 3.64 (3.64-3.64) |
| **Anesthesia related factors** |  |  |
| Anesthesia duration |  |  |
| ≤ 90 minutes | Ref | Ref |
| 90~120 minutes | 1.46 (1.14-1.87) | 0.93 (0.79-1.10) |
| 121~180 minutes | 2.03 (1.62-2.55) | 0.98 (0.84-1.15) |
| >180 minutes | 2.75 (2.18-3.47) | 1.31 (1.12-1.53) |
| Combined intravenous and inhalation anesthesia | Ref | Ref |
| Total intravenous anesthesia | 1.35 (0.81-2.27) | 1.24 (0.95-1.63) |
| Inhalation anesthesia | 0.24 (0.06-1.02) | 0.91 (0.52-1.60) |
| **Postsurgical events** |  |  |
| Admission to ICU (yes vs no) | 1.74 (0.47-6.51) | 1.25 (0.85-1.83) |
| Any Postoperative complications ^2^ (yes vs no) | 2.86 (2.05-4.00) | 1.55 (1.30-1.85) |

^1^ OR and 95%CI were derived from generalized liner mixed-effect models, adjusted for age, sex, education, BMI, smoking, and drinking.

^2^ Included pulmonary complication, major adverse cardiac event, acute kidney injury, and infection.

CPSP: chronic postsurgical pain, pain in the surgical area persisted for more than 3 months after surgery

POCD, postoperative cognitive dysfunction, indexed by AD-8 score ≥ 2
